# Physical, Mechanical, and Structural Properties of the Polylactide and Polybutylene Adipate Terephthalate (PBAT)-Based Biodegradable Polymer during Compost Storage

**DOI:** 10.3390/polym15071619

**Published:** 2023-03-24

**Authors:** Dmitry Myalenko, Olga Fedotova

**Affiliations:** All-Russian Dairy Research Institute, Lyusinovskaya Street, 35, 7, 115093 Moscow, Russia

**Keywords:** composting, polylactide, polybutylene adipate terephthalate, Fourier transform infrared spectroscopy (FTIR), scanning electron microscopy (SEM)

## Abstract

Today, packaging is an integral part of any food product, preserving its quality and safety. The use of biodegradable packaging as an alternative to conventional polymers reduces the consumption of synthetic polymers and their negative impacts on the environment. The purpose of this study was to analyze the properties of a biodegradable compound based on polylactide (PLA) and polybutylene adipate terephthalate (PBAT). Test samples were made by blown extrusion. The structural, physical, and mechanical properties of the PLA/PBAT material were studied. The property variations during compost storage in the lab were monitored for 365 days. The physical and mechanical properties were measured in accordance with the GOST 14236-2017 (ISO 527-2:2012) standard. We measured the tensile strength and elongation at rupture. We used attenuated total reflectance Fourier transform infrared microscopy (ATR-FTIR) to analyze the changes in the material structure. This paper presents a comparative analysis of the strengths of a biodegradable material and grade H polyethylene film (manufactured to GOST 10354-82). PLA/PBAT’s longitudinal and transverse tensile strengths are 14.08% and 32.59% lower than those of LDPE, respectively. Nevertheless, the results indicate that, given its physical and mechanical properties, the PLA/PBAT material can be an alternative to conventional PE film food packaging. The structural study results are in good agreement with the physical and mechanical tests. Micrographs clearly show the surface deformations of the biodegradable material. They increase with the compost storage duration. The scanning microscopy (SEM) surface analysis of the original PLA/PBAT films indicated that the PLA structure is similar to that of a multilayer shell or sponge, which is visible at medium and especially high magnification. We conclude that PLA/PBAT-based biodegradable materials are potential substitutes for conventional PE polymer films.

## 1. Introduction

The wide range of polymer packaging in use has led to serious problems with the proper recycling and disposal of large-scale polymer waste generated at the end of the packaged product lifecycle, negatively affecting the environment [1]. With depleting fossil resources and a deteriorating environment, we should re-think natural resource extraction.

The United Nations [1] and its partners and European research and technology organizations (RTOs) [2] believe that the climate, environmental protection, and the efficient utilization of natural resources are highly important for building a sustainable, circular economy. One trend is using alternative, biological raw materials for the production of biodegradable polymers [3].

To prevent polymer waste accumulation outdoors, plastic products should be recyclable or biodegradable [4]. There is high demand for biodegradable polymers that quickly decompose when exposed to the elements and bacteria but have consumer properties similar to those of conventional polymers [5]. Using the proper materials, we can manufacture highly flexible films with high impact toughness and elongation at break (more than 500%), good fusibility (melting point at 115–125 °C), and good biodegradability [6,7].

There are technologies available to minimize waste, such as low-waste manufacturing [8], biodegradable materials and compositions [9], advanced waste collection, sorting, and recycling processes [10], polymer production waste recycling, etc. [11]. Today, 80% of the polymer waste is polypropylene (PP), polyethylene (PE), and polyethylene terephthalate (PET). The chemical properties of these materials are such that these substances are not suitable for composting and are resistant to microorganisms that could otherwise accelerate the decomposition.

The most promising materials are polylactic acid (PLA), polybutylene succinate (PBS), polybutylene succinate adipate (PBSA), polyhydroxyalkanoate (PHA), polybutylene adipate terephthalate (PBAT), polyhydroxybutyrate-co-hydroxyvalerate (PHBV), and their compounds [12,13,14,15]. The biodegradation rate greatly depends on the ambient temperature and humidity [8]. For example, PLA shows a high biodegradation rate at temperatures well above room temperature. This makes the substance very suitable for industrial-grade composting. Although its biodegradation rate in soil and bodies of water is higher than those of PE, PP, and PET, it can still accumulate in such media for several decades [16]. Other biodegradable polymers, such as PBS and PBSA, have much higher biodegradation rates in soil than PE, PP, and PET polymers, although they are generally not certified as such because their lifecycle durations in food packaging do not always meet certification standards [17]. In this regard, PHA and PBAT are among the few polymers that are certified as biodegradable in all environments, including marine, and therefore, they have great utilization potential [18,19]. Scientists note the prospects of using PHBV as a biodegradable polymer in the medical industry and as a basis for the creation of composites [14,15,20,21]. Pavel Brdlík and his colleagues studied the possibility of the biodegradation of a material based on PHBV CaCO_3_ and the natural plasticizer acetyl tributyl citrate (ATBC) in various ratios. The results showed the prospects of such materials. The rate of biodegradation increased to 90% within 90 days, depending on the composting conditions [15].

Polymer manufacturing innovations can lead to more sustainable [22,23] medical products [24,25] and packaging [26], as these are the two primary uses of synthetic polymers. Studies of PLA [12,13,27] and PBAT [1,6,28] have focused on the properties and structures of biodegradable materials, while the applications of such materials as food packaging are not well investigated. It should be noted that many polyesters cannot be used as dairy and food product packaging because of their properties. They are applied as coatings on biodegradable polymer substrates, increasing the resistance to grease and moisture. Such materials are suitable for packaging any product, even with >15.0% humidity [29,30,31].

Scientists from all over the world are engaged in the development of biodegradable packaging materials for food [32,33,34,35]. The use of biodegradable materials for the production of disposable tableware, plates, spoons, cups, and other products has already been successfully implemented in various countries. For example, PBAT and aliphatic PCL are used in the creation of films (mulch and containers), filaments, thermoformed and injection-molded products, blown bottles, and packaging materials for products with a short service life, as well as for the production of biodegradable disposable food bags and other bags [35,36,37,38,39,40]. However, it should be noted that there is no ideal solution for food packaging at the moment. Food products are complex systems that require a set of special physico-chemical, microbiological, sanitary and hygienic, and operational characteristics, and based on this, the use of biopolymers can be significantly limited [33,36,37,38,40]. For dairy products, this problem is even more urgent, since milk is a living composite system with different acidity and complex biochemical properties, which, during storage, can have a negative impact on the safety of the product in biodegradable compositions [41].

The key purpose of polymer packaging is the preservation of dairy and food products throughout their life cycle, which is necessary [11,42,43,44,45,46]. The packaging should also be environment-friendly. It should be noted that the introduction of various components into the polymer can be a risk factor for packaged product safety [42,44,45,47]. This is especially true for special diets and baby food [41,42,44,46]. Another point of interest is functional packaging [48,49,50,51]. This is especially promising for solid food products. Making functional packaging from polymer films will reduce the risk of contamination during packaging and preserve the product throughout its entire lifecycle [52,53,54,55].

The purpose of this study was to study the changes in the physico-mechanical and morphological properties of a biodegradable compound material based on PLA and PBAT during compost storage and its potential use as packaging for milk and dairy products. 

## 2. Materials and Methods

### 2.1. Polybutylene Adipate Terephthalate

**Figure 1:**
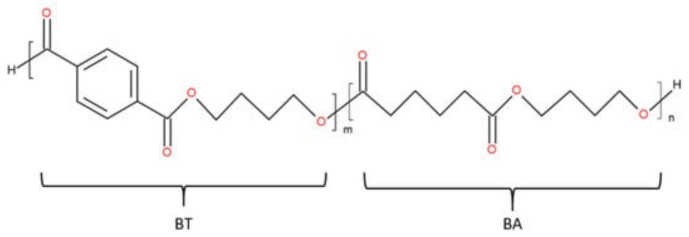
PBAT chemical structure (m, n—number of repeating links).

PBAT is a fully biodegradable polyester with two types of dimers: BT and BA (Figure 1). The first dimer forms a rigid matrix consisting of 1,4-butanediol and terephthalic acid monomers. The other dimer is the flexible part consisting of 1,4-butanediol and adipic acid monomers [56], resulting in high flexibility and elasticity [56]. BASF, the largest manufacturer (Ludwigshafen, Germany), makes PBAT under the Ecoflex^®^ brand name (e.g., Ecoflex F Blend C1200). This polymer has a 1.26 g/mol density, 52.1 kg/mol number average molecular weight (M n), and a 2.0 polydispersity index.

### 2.2. Polylactic Acid (Polylactide)

Polylactic acid or polylactide (PLA) is one of the most important biodegradable polymers. PLA has been extensively studied for a wide range of applications, such as disposable household items, food packaging, agricultural films, drug delivery systems, and implantable medical devices [57,58,59].

**Figure 2:**
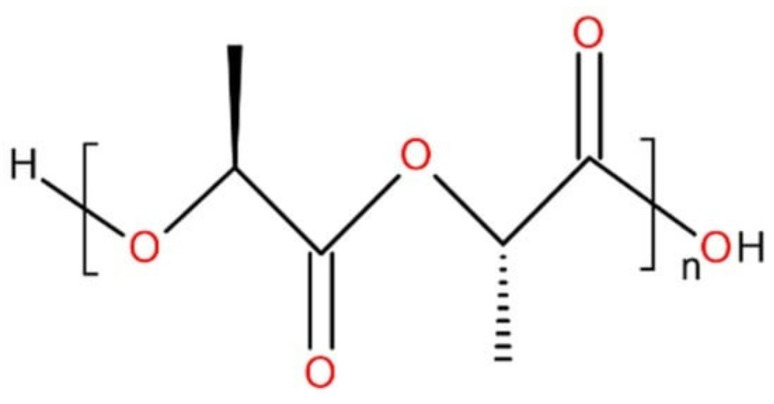
PLLA chemical structure (n—number of repeating links).

PLA is a biodegradable and renewable aliphatic polyester [60]. It is made by the direct polycondensation of lactic acid. PLA can also be obtained by the ring-opening polymerization of cyclic lactide. PLA has two optically active and crystallizable isomeric forms: PDLA and PLLA (Figure 2).

A previous study [61] notes that the l-to-d isomer ratio affects PLA’s final properties. A statistically organized poly(DL-lactide) (PDLLA) complements such pure PLA stereoisomers as poly(l-lactide) (PLLA) and poly(d-lactide) (PDLA). PDLLA is usually amorphous because it lacks the chain regularity required for crystallization. Pure PLLA and pure PDLA are semi-crystalline. LA has some promising properties as a food packaging material, such as the high modulus of elasticity, strength, and transparency (in its amorphous state). It is also easy to manufacture [58]. However, it has some disadvantages, such as high brittleness, a low deformation temperature, and a low crystallization rate. These could be critical for certain types of product packaging [62,63,64].

NatureWorks (Minnetonka, MN, USA), a major manufacturer, produces PLA under the Ingeo™ brand name (e.g., Ingeo™ Biopolymer 2003D). Its density is 1.24 g/mol, its number average molecular weight (M n) is 127.0 kg/mol, its polydispersity index is 1.6, and the D-isomer content is approximately 4.4%.

For tests, PBAT/PLA mixtures are often prepared by melt mixing. Interetherification can occur between the two polyesters at elevated temperatures when interacting for long periods, resulting in increased bendability [65].

The biodegradation of polymers is a complex process with several stages [66]. In the initial stage of PLA degradation, the polymer is split into monomers or low-molecular-weight oligomers, where the ester bonds are hydrolytically degraded. The effect of microorganisms on composting leads to a great reduction in the molecular weight of the material [66,67,68]. PLA composting is more efficient in combined hydrolysis and microbial activity processes [69]. The elevated temperatures during composting also accelerate PLA hydrolysis, especially above 50 °C [70]. Polymer mixtures have different morphologies depending on their composition. It also affects the biodegradation process [71]. Additionally, different biodegradable polymers decompose at different rates [72,73]. A previous study [73,74] showed that PLA/PBAT mixtures decompose at a lower rate than PLA or PBAT. As noted above, several factors affect the decomposition rate, such as the temperature, the moisture content of the compost, and its microbiological composition [75,76,77].

### 2.3. PLA/PBAT Mixture-Based Biodegradable Films

The materials we used are polymeric biodegradable films based on a PLA/PBAT mixture (CO-PLAS Bio 1002) manufactured by Eurotex, Timashevsk, Russia. This substance is a biopolymer compound compliant with GOST EN 13432-2015 (IDT EN 13432:2000) with high thermal stability, strength, and gas permeability. The thickness of the biodegradable film was 20 microns.

The CO-PLAS Bio 1002 biopolymer compound was processed with the standard blown film extrusion process and equipment and a standard LDPE head. The temperature was 140–165 °C.

We believe that the materials we investigated can be widely used in the dairy and food industry as packaging materials in many form factors and can become a good alternative to conventional polymers.

### 2.4. Composting Conditions

A study of changes in the degradation rate of the tested film materials was carried out under laboratory conditions without the use of industrial composting. The temperature conditions of the experiment were 20 ± 2 °C, with humidity of 65–80%. Under the above conditions, layering was carried out in compost, consisting of classical soil and a composting accelerator, which includes at least 30% wheat bran, sodium bicarbonate, and 5% soil microorganisms.

To simulate composting conditions, a soil mixture with the following composition was used: nitrogen—at least 230 mg/L; phosphorus—at least 300 mg/L; and potassium—at least 350 mg/L.

### 2.5. Methods

We tracked the weight changes with an AND GR-300 analytical balance, Special I accuracy class (GOST 24104-01).

We measured tensile strength (MPa) and elongation at break (%). For longitudinal and transverse tests, we used a Shimadzu EZ-LX universal testing machine compliant with GOST 14236-2017 (ISO 527-2:2012). The max range of the load cell is 2000 N, and the stroke is 920 mm. The TRAPEZIUM X software processed the measurements. The number of conducted series of the experiment was 3; the number of repetitions in the series was 10.

We estimated the particle size with bright-field microscopy using an Axio Lab.A1 transmitted light optical microscope with an Axiocam 105 color camera at 1000× final magnification. The IR spectra of the PLA/PBAT composite samples were recorded with a Bruker Lumos FTIR spectrometer and microscope macromodule in the 4000–600 cm^−1^ range. The samples were analyzed with a Vega 3 scanning electron microscope (Tescan, Czech Republic). Before the examination, the samples were platinum-coated to a ~20 nm thickness.

## 3. Results

**Figure 3:**
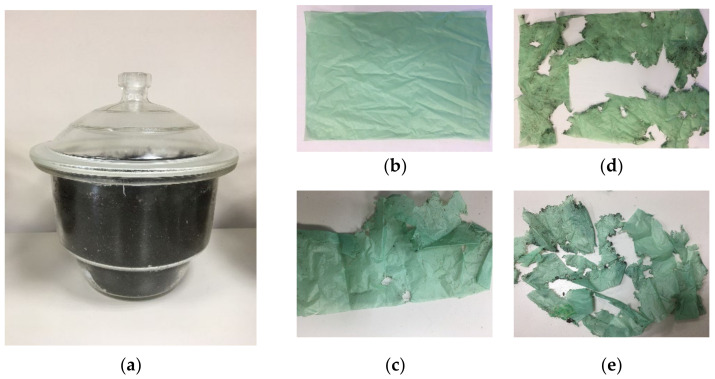
PLA/PBAT-based biodegradable film samples in compost: (**a**) the compost chamber, (**b**) the PLA/PBAT sample before the test, (**c**) the PLA/PBAT sample after soil storage for 90 days, (**d**) the PLA/PBAT sample after soil storage for 180 days, (**e**) the PLA/PBAT sample after soil storage for 365 days.

The appearances of a biodegradable film sample before and after the compost storage are shown in Figure 3.

As we can see in Figure 3, the biodegradable film sample is highly degraded after composting for 90 days. This is confirmed by the changes in the weight of 1 m^2^ of the sample, tensile strength, and elongation at break, as presented in Table 1, which shows changes in the weight of 1 m^2^ of the PLA/PBAT biodegradable material after composting for 90 days.

The table shows that the 1 m^2^ weight greatly decreases soon after the very beginning of the experiment. The weight drop is 7.4% at 30 days of storage, 14.8% at 60 days, and 29.6% at 90 days. We analyzed the results of 20 repeated measurements at each storage stage.

This is because soil microorganisms break the PLA macromolecules and form identical size chains. Subsequently, an equilibrium is reached, when the decomposition is relatively slow.

Many factors may intensify this process. For example, heating PLA initiates the thermochemical destruction of chemical bonds, which reach their equilibrium value. With this feature, the thermodestruction process can be defined analytically with the Arrhenius equation, which accounts for the thermal effects on the polymer bond decay rate [74,78].

As reported in the available sources, depending on the storage conditions and composition, the weight change can range from 25% to complete decomposition in 90 to 545 days of compost storage [78]

By analyzing the results, we can conclude that the composting storage of the polymer samples results in weight changes, but these changes depend significantly on the structure and composition of the material, and some external factors.

Food systems, particularly milk-based foods, are an extremely “capricious” object of packaging; therefore, the basic problem of the new biodegradable packaging of dairy products is to ensure and confirm the stability of such packaging in the full process from its production to the consumer’s table, since modern packaging is an integral component of food products. Among the criteria that allow us to assess the presence of changes in the packaging material with traceability of the packaging life cycle are physical and mechanical indicators. Polyethylene films, including those for dairy products, have been comprehensively studied [79,80,81,82,83,84,85]; the authors of this article obtained data on changes in (or the immutability of) their properties under natural and critical conditions, for example, when exposed to low temperatures and ultraviolet radiation [86,87].

In the present study, they were used for a comparative assessment of the initial physical and mechanical parameters of samples of the studied materials. It is shown that, in general, the levels of the obtained indicators are sufficient for the use of the selected biodegradable films as packaging.

Before the compost storage of the PLA/PBAT biodegradable material samples, we tested their physical and mechanical properties and compared them with those of conventional PE-based polymers. We plotted the physical and mechanical property changes vs. the changes in the force (H) applied to the samples at the moment of failure and the strain (relative elongation at rupture) of the samples, expressed in %. The results are shown in Table 2. 

The data indicate that the strain variations in the PE film and the PLA/PBAT-based film are identical. The force vs. elongation curve features the three deformation stages typical for the given polymer: elastic (reversible) deformation; necking; and oriented polymer deformation.

The longitudinal tensile strength of the PE film and the PLA/PBAT-based biodegradable film is 32.59% less than that of the LDPE film and increases by 14.08% in the transverse direction. The relative elongation at rupture in the longitudinal direction is increased by 8.8%, and in the transverse direction, it is decreased by 59.40%. It should be noted that the values for all the polymer film samples are compliant with regulations (GOST 10354-82 for grade H polyethylene films). The significant variation in elongation values can be eliminated as required for specific product packaging by adding flexibilizers that increase the elasticity or organic modifiers [88,89,90].

**Figure 4:**
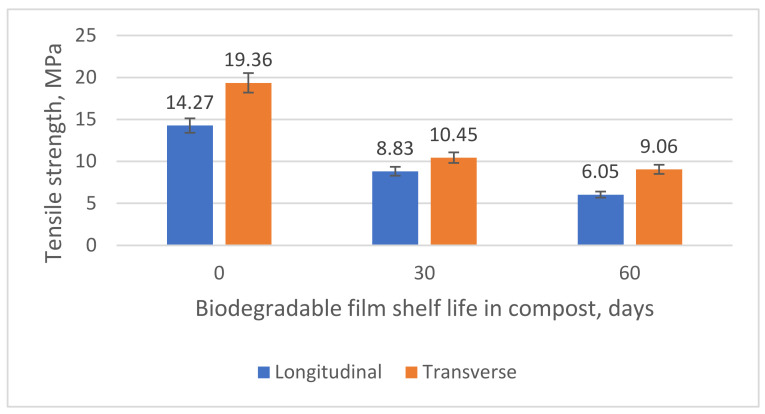
Changes in the tensile strength (MPa) of the biodegradable PLA/PBAT-based film after composting for 60 days.

**Figure 5:**
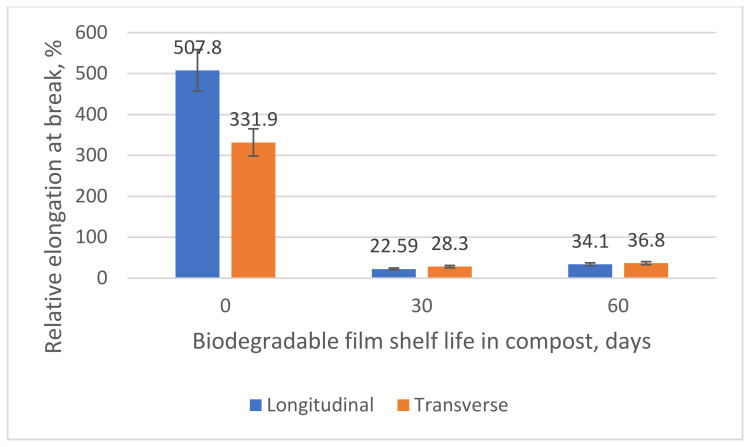
Changes in the relative elongation at break (%) of the biodegradable PLA/PBAT-based film after composting for 60 days.

We tracked the changes in the physical and mechanical parameters (δ, MPa; ε, %) to estimate the PLA/PBAT-based biodegradable film destruction rate variations. The results are shown in Figure 4 and Figure 5.

The results show that the physical and mechanical properties of the samples decrease sharply relative to their original values. Table 3 lists the results. The values are in good agreement with the available data [91,92].

The data show that after 60 days of composting, the tensile strength of the PLA/PBAT-based material decreases by more than 50% compared to the reference samples. The relative elongation at break decreases almost twofold. By comparing the results with the weight changes, we can summarize them as follows: PLA/PBAT-based materials have good properties that result in accelerated biodegradation.

**Figure 6:**
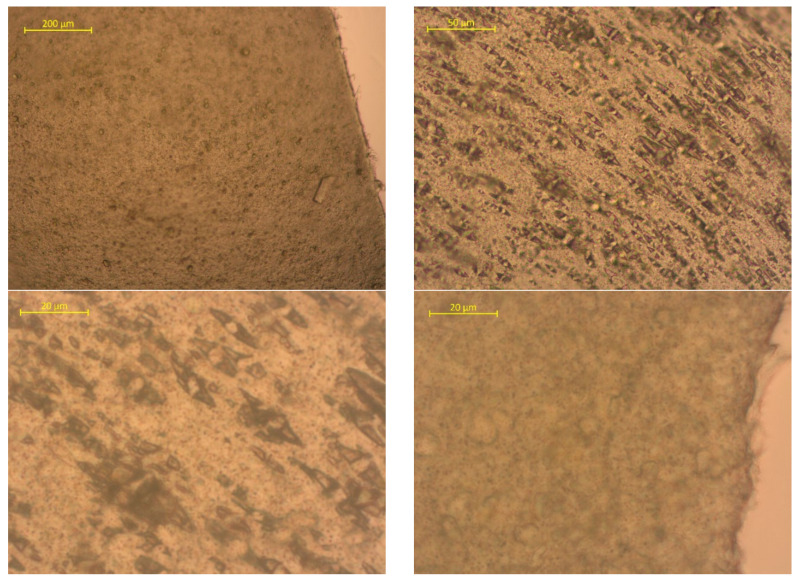
Surface photos of the PLA/PBAT material before composting (various magnifications).

**Figure 7:**
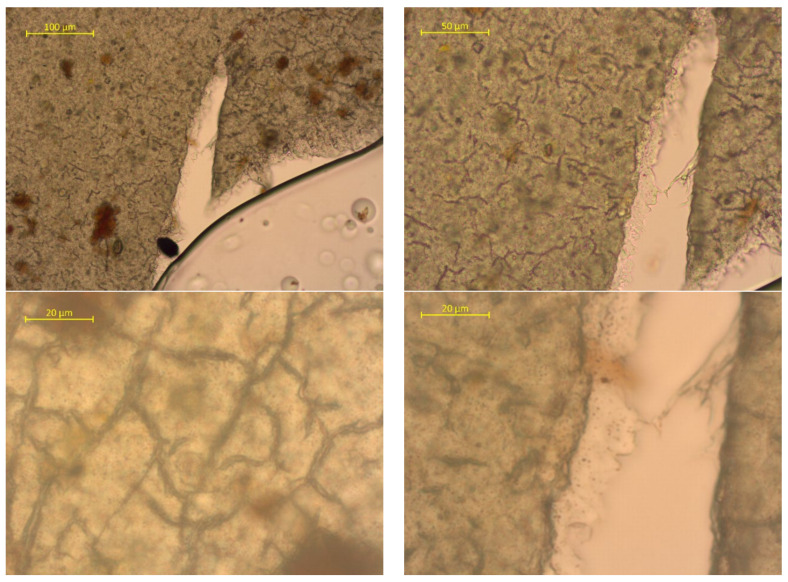
Surface photos of the PLA/PBAT material after composting for 180 days (various magnifications).

**Figure 8:**
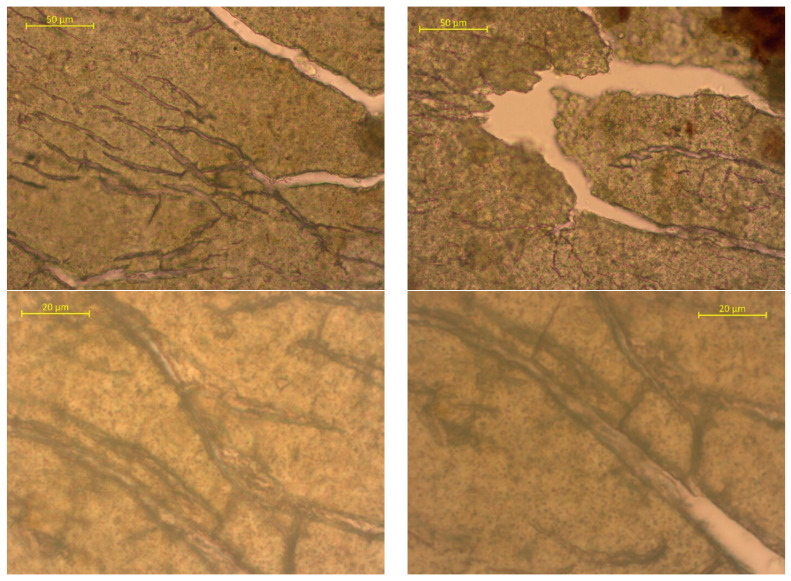

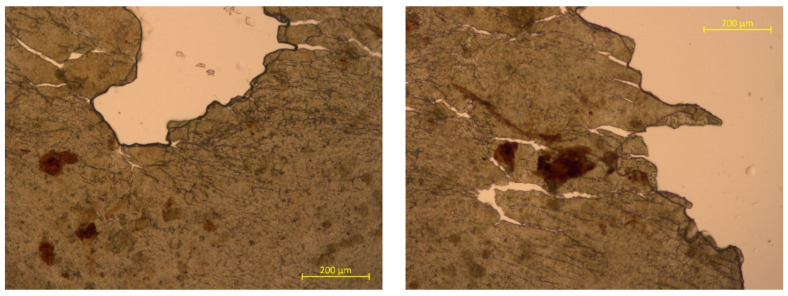
Surface photos of the PLA/PBAT material after composting for 365 days (various magnifications).

To assess the surface changes, we performed microscopic studies on the PLA/PBAT film after composting. The photos are shown in Figure 6, Figure 7 and Figure 8.

The photos show that the material is a mixed heterogeneous compound. The PLA particles are crystalline, while the PBAT particles are more dispersed and have no ordered structure. The small dark inclusions are the pigment. There are no signs of mechanical degradation on the surface before composting.

The surfaces feature multiple cracks. Note that the cracks are distributed unevenly. This may be attributed to the initially irregular structure of the composite. The fine PBAT particles are more susceptible to degradation after composting in natural conditions (20 °C, RH under 80%). The surface cracks are not through-cracks. The lateral slice has translucent areas, indicating a density decrease. There may be several reasons for this: oxidation, microorganism activity, etc.

**Figure 9:**
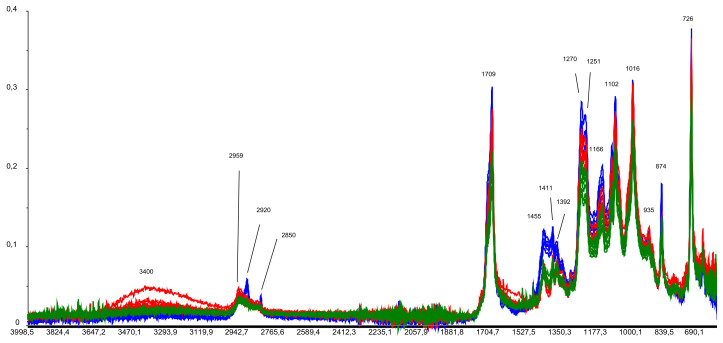
IR spectra of the PLA/PBAT composite samples in the 4000–600 cm^−1^ range (blue: before composting; red: after 180 days of composting; green: after 365 days of composting).

The surface features multiple cracks. The higher-magnification images show transparent cracks and some through-cracks. The comparison of the samples after 180 and 365 days of composting indicates a significant increase in the number of cracks on virtually the entire surface. Through-holes can be detected even at the lowest magnifications. The dark spots in the photos are the residual soil/compost. The results are in good agreement with the physical and mechanical test results. Figure 9 shows the results of spectroscopic tests with Fourier transform infrared spectroscopy (FTIR).

Multiple spectra were obtained for each sample: 5 before composting, 6 after 180 days of composting, and 8 after 365 days of composting, for a total of 19 spectra.

The absorption bands at 2920 cm^−1^ and 2850 cm^−1^ are blurred after 180 days of composting. A 2959 cm^−1^ band appears after 365 days.

The spectra demonstrate significant differences in the 1460 to 1380 cm^−1^ range. The intensity of the bands in this range decreases as the composting period increases. Presumably, composting destroys some bonds, as indicated in the table for the 1455, 1411, and 1392 cm^−1^ bands (Table 4).

Additionally, the intensity of the narrow peaks between 1300 and 1000 cm^−1^ and at 1709 cm^−1^ decreases with the composting time.

The IR spectrum of the composted PLA/PBAT film in the entire 690 to 4000 cm^−1^ range shows strong deviations from the reference sample spectrum. This indicates the destructive processes occurring in this compound, especially after 365 days of composting. The data presented in [78,93,94] also reveal significant fluctuations in the bonds of groups, which clearly describes the course of destructive processes.

**Figure 10:**
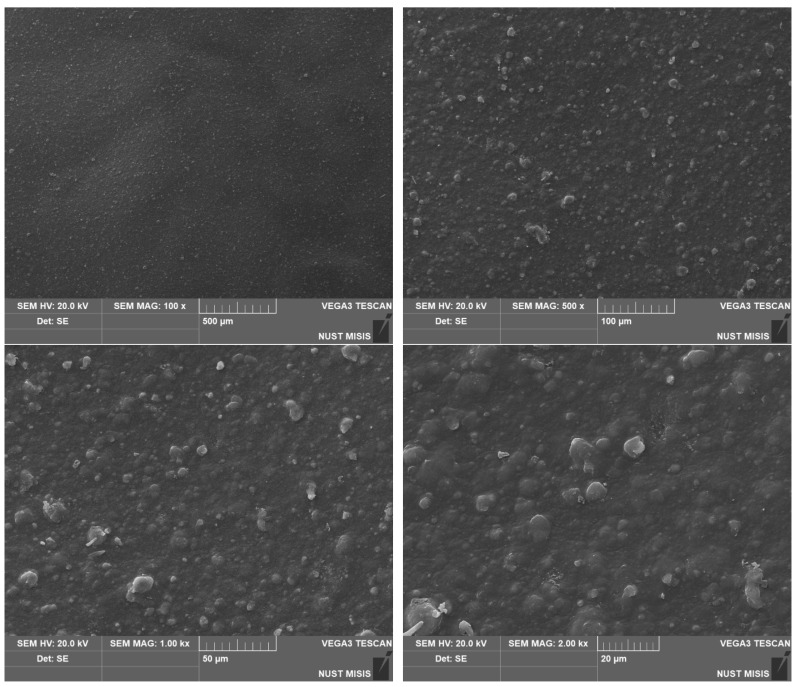

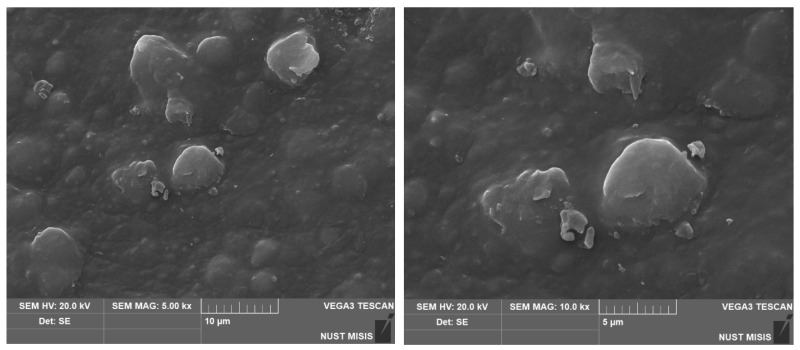
SEM images of the PLA/PBAT film surface before composting.

**Figure 11:**
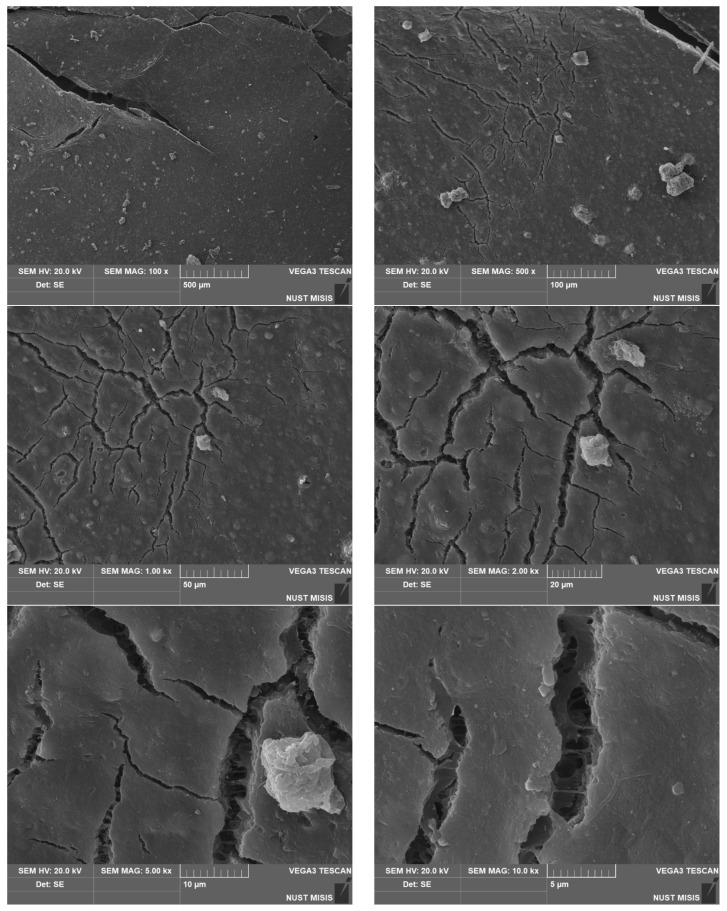
SEM images of the PLA/PBAT film surface after composting for 180 days.

**Figure 12:**
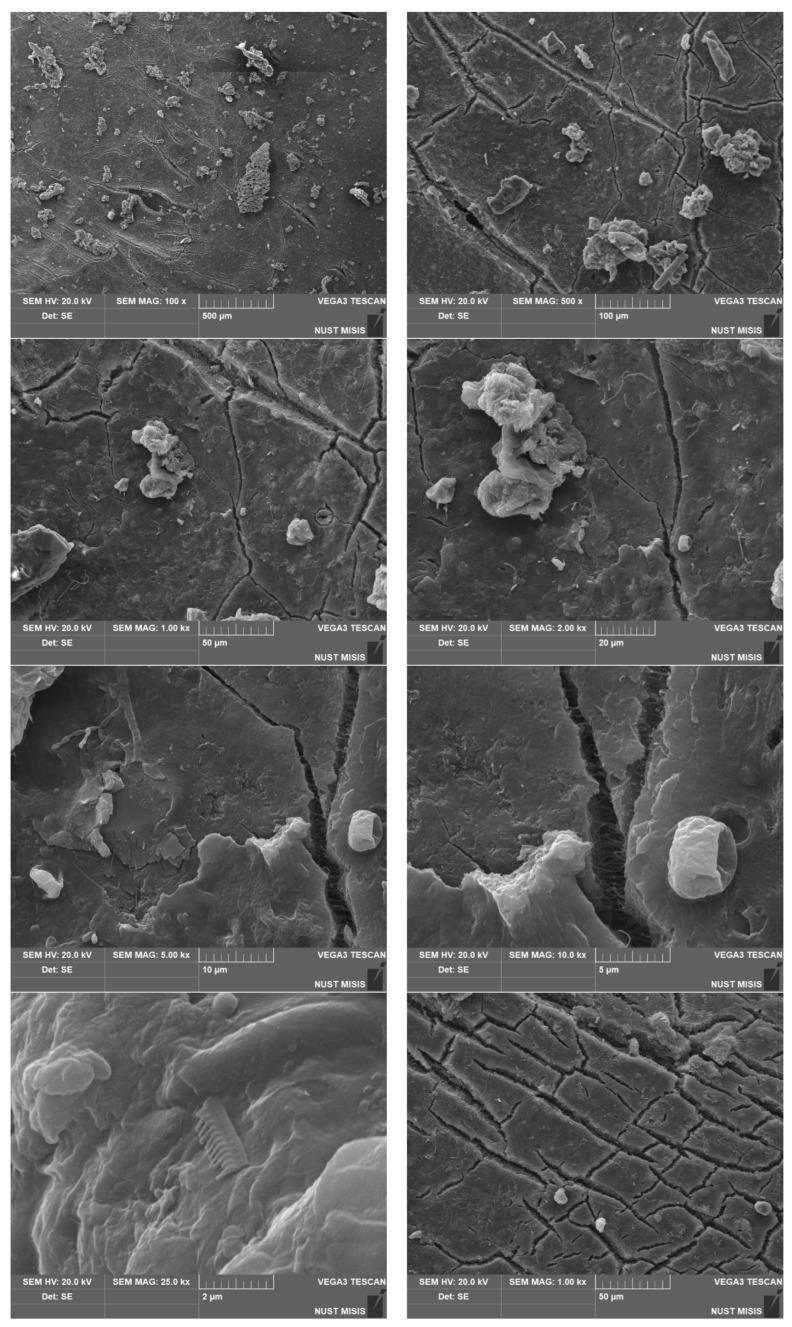

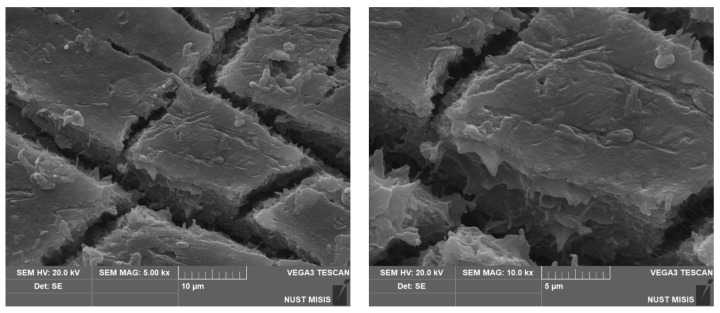
SEM images of the PLA/PBAT film surface after composting for 365 days.

We performed microstructural studies to evaluate the surface changes in the biodegradable material samples after composting. Figure 10, Figure 11 and Figure 12 present PLA/PBAT surface photos taken with a scanning electron microscope.

The surface analysis shows significant changes in the surface structure after composting for 365 days (Figure 9, Figure 10 and Figure 11).

The structural studies before composting indicate that higher magnification reveals noticeable microcracks that do not go deep into the material and do not affect its strength, as confirmed by the physical and mechanical tests. No significant changes in the structure of the surface layers were observed.

After composting for 180 days, there are noticeable, significant changes. The surface has numerous cracks going deep into the body. The cracks are micellar due to the material heterogeneity.

At 10,000× magnification, translucent areas around the cracks and fractures become noticeable. This indicates a decrease in the density, which can be attributed to oxidation, microorganism activity, etc. According to the available literature, the extensive degradation of PLA begins at elevated humidity and temperatures above 50 °C, while PBAT begins to decompose at lower temperatures (20 °C and RH 80%). At 5000× magnification, the surface shows depressions and pits. This indicates the different degradation rates of the film components. The data obtained correlate well with the results of physical and mechanical tests and with data reported by scientists who conducted similar studies [78,95]. Depending on the composting conditions and the composition of polymer compost, the dynamics of biodegradation differ, but the general trend persists [93,94,95].

After composting for 365 days, the degradation is significantly accelerated: there are cracks and delamination over the entire surface.

## 4. Conclusions

The physical and mechanical properties of the biodegradable film deteriorate dramatically after 60 days of composting. There is a 53.26–57.60% decrease in the tensile strength and an 88.91–93.28% decrease in the relative elongation at break (depending on the direction). The relative elongation at break decreases almost twofold. 

Microscopic studies indicate that, after 365 days of composting, the surface has a large number of cracks and through-holes on virtually the entire surface. The data are in good agreement with the physical and mechanical test results.

The analysis of the IR spectra of the PLA/PBAT material before and after composting showed that the entire 690 to 4000 cm^−1^ range strongly deviates from the reference sample spectrum. Significant spectral differences were found in the 1460 to 1380 cm^−1^ range. The intensity of the bands in this range decreases with the progression of the composting period. The peaks indicate destructive processes in the compound, especially after 365 days of composting.

The scanning electron microscopy examination of the surface after 365 days of composting revealed significant changes. The biodegradable film surface features numerous cracks going deep into the material on virtually the entire surface.

The dynamics of changes in the physical and mechanical parameters of the polymer biodegradable film indicate a relatively small time range in which these materials can be used as packaging for dairy products. These may be fermented milk products, such as cottage cheese, with a limited shelf life.

The obtained complex data on the changes in the structures of the surfaces of the samples under study indicate the dynamic processes of their decomposition, which creates the real prospect of minimizing environmental risks for the purpose of environmental protection associated with the disposal of packaging.

## Figures and Tables

**Table 1:** Experimental results: 1 m^2^; weight, g.

Sample No.	Experimental Results: 1 m^2^; Weight, g			
	0 Days	30 Days	60 Days	90 Days
Average	2.7	2.5	2.3	1.9
Weight drop, %	0	7.4	14.8	29.6

**Table 2:** Physical and mechanical test results.

	Actual Results			
	PE Film		PLA/PBAT Film	
	Longitudinal	Transverse	Longitudinal	Transverse
δ, MPa	21.17	16.97	14.27	19.36
ε, %	466.60	817.63	507.8	331.9

**Table 3:** Decrease in the physical and mechanical properties of the biodegradable PLA/PBAT-based film after soil storage.

Property	Soil Storage Period, days	
	30	60
Reduction in δ (MPa) relative to the reference sample, %:		
Longitudinal	38.12	57.60
Transverse	46.02	53.26
Reduction in ε (%) relative to the reference sample, %:		
Longitudinal	95.55	93.28
Transverse	91.47	88.91

**Table 4:** IR spectral bands.

Band, cm^−1^	Functional Group
3400	OH
2959	C-O
2920	CH_3_
2850	CH_2_
1710	C=O
1455	Phenylene (C_6_H_4_)
1411	C-H
1392	-C(CH_3_)_3_
1270	CH in CH_2_ and CH groups *
1251	CH in CH_2_ and CH groups *
1166	C-O
1102	C-O-C
1016	Phenylene (C_6_H_4_)
935	C-O *
874	C=O
726	CH_2_

* Reference data [66,88].

## Data Availability

Not applicable.
